# Supplementary material for: Psychometric properties of End Stage Renal Disease-Adherence Questionnaire-Sinhalese version among patients receiving haemodialysis
Source: PLoS One. 2023 Oct 20;18(10):e0292938. doi: 10.1371/journal.pone.0292938 (PMC10588851; doi:10.1371/journal.pone.0292938)
Supplement: S1 File — (PDF) [file pone.0292938.s001.pdf]

වකුගඩු රෝගයේ අවසාන අවධියේ පසුවන රෝගීන්ගේ ප්‍රතිකාර අනුකූලතාවය සොයා බැලීම පිළිබඳ ප්‍රශ්නාවලිය

මෙම සමීක්ෂණය මගින් ඔබ ඔබේ රුධිර කාන්දුකරණ ප්‍රතිකාර කාලසටහන කෙතරම් හොඳින් අනුගමනය කරන්නේද යන්න සහ ඖෂධ, ආහාර ගැනීම සහ දියර සීමා කිරීම සම්බන්ධ වෛද්‍ය නිර් දේශ අනුගමනය කිරීම පිළිබඳව ඔබේ අදහස විමසයි. ඔබේ රුධිර කාන්දුකරණ ප්‍රතිකාරය, ඖෂධ, දියර සීමා කිරීම සහ නිර් දේශිත ආහාර වේල අනුගමනය කිරීම ඔබට අපහසු දැයි තේරුම් ගැනීමට මෙම තොරතුරු අපට උපකාර කරයි. කරුණාකර සුදුසු කොටුව සලකුණු කිරීමෙන් සෑම ප්‍රශ්නයකටම පිළිතුරු දෙන්න. පිළිතුරු දිය යුතු ආකාරය පිළිබඳව ඔබට විශ්වාසයක් නොමැති නම්, කරුණාකර ඔබට අදාළ වන හොඳම පිළිතුර තෝරන්න.

**I.**	**සාමාන්‍ය තොරතුරු**		
01.	ඔබ රුධිර කාන්දුකරණ ප්‍රතිකාර ක්‍රමය(ලේ පිරිසිදු කිරීම) ආරම්භ කල දිනය හෝ නවත්වා නැවත ආරම්භ කල දිනය		මුලින්ම පටන්ගත් දිනය/ නවත්වා නැවත ප්‍රතිකාර පටන්ගත්තා නම්, දිනය<br>.....................<br>මාසය/වර්ෂය
02.	ඔබ කවදා හෝ අන්ත්‍රාවරණ කාන්දුකරණ ප්‍රතිකාරය (peritoneal dialysis) ලබා තිබේද?		නැත.(1)<br>ඔව්.(2)<br>පිළිතුර ඔව් නම්,<br>..................... ....<br>මාසය/වර්ෂය සිට මාසය/වර්ෂය
03.	ඔබට වකුගඩු බද්ධ කිරීමක් කර තිබේද?		නැත.(1)<br>ඔව්. (2)<br>පිළිතුර ඔව් නම්,<br>කොපමණ වතාවක්ද?...........................
04.	රුධිර කාන්දුකරණ (ලේ පිරිසිදු කිරීමේ) මධ්‍යස්ථානය වෙත යාම සඳහා ඔබ කුමන ආකාරයේ ප්‍රවාහන පහසුකමක් භාවිතා කරන්නේද?		☐ පෞද්ගලික ප්‍රවාහන සේවයෙනි.(1)<br>☐ බස්රථයෙනි.(2)<br>☐ කුලී රථයෙනි.(3)<br>☐ ගිලන්රථ මගිනි.(4)<br>☐ වෙනත්(සඳහන් කරන්න)(5) ....
05.	රුධිර කාන්දුකරණ (ලේ පිරිසිදු කිරීමේ) මධ්‍යස්ථානය වෙත ඔබ සමඟ පැමිණෙන්නේ කවුද?		☐ මා විසින් තනිවම පැමිණෙමි.(1)<br>☐ දෙමව්පියන් සමඟය.(2)<br>☐ සහකරු (බිරිඳ/ස්වාමියා) සමඟයි.(3)<br>☐ දරුවා සමඟයි.(4)<br>☐ යහළුවෙකු සමඟයි.(5)<br>☐ වෙනත් (පුද්ගලයා සඳහන් කරන්න.)(6).....................

**II.** රුධිර කාන්දුකරණ ප්‍රතිකාරය/ ලේ පිරිසිදු කිරීමේ ප්‍රතිකාරය			
06.	සතියකට කොපමණ වාර ගණනක් ඔබ රුධිර කාන්දුකරණ (ලේ පිරිසිදු කිරීමේ) ප්‍රතිකාරය ලබාගන්නේද?		☐ දින 1(1)<br>☐ දින 2(2)<br>☐ දින 3(3)<br>☐ දින 4(4)<br>☐ දින 4ට වඩා වැඩි(5)
07.	රුධිර කාන්දුකරණ (ලේ පිරිසිදු කිරීමේ) එක් ප්‍රතිකාරයක් සඳහා ඔබ කොපමණ පැය ගණනක් ගත කර ඇත්ද?		☐ පැය 3ට වඩා අඩු(1)<br>☐ පැය 3(2)<br>☐ පැය 3 විනාඩි 30(3)<br>☐ පැය 4(4)<br>☐ පැය 4ට වඩා වැඩි(5)<br>☐ වෙනත් (සඳහන් කරන්න)(6)
08.	ඔබගේ රුධිර කාන්දුකරණ (ලේ පිරිසිදු කිරීමේ) ප්‍රතිකාර කාලසටහන ඔබට පහසුද? (කරුණාකර ඔබට අදාළ වන නිවැරදිම පිළිතුර තෝරන්න.)		☐ ඔව්.(1)<br>☐ නැත, මා ඉතාමත් වේලාසනින් ප්‍රතිකාර මධ්‍යස්ථානයට පැමිණිය යුතු නිසා.(2)<br>☐ නැත, මාගේ දෛනික කාලසටහනට නොගැලපෙන නිසා.(3)<br>☐ නැත, මාහට රෝහලෙන් ලබාදෙන වෙලාවට පැමිණිය යුතු නිසා(4).<br>☐ නැත, මට නිවසේ සිට රෝහලට පැමිණීමට බොහෝ වෙලාවක් ගතවන නිසා(5).<br>☐ වෙනත් ....(6)
09.	රුධිර කාන්දුකරණ (ලේ පිරිසිදු කිරීමේ) ප්‍රතිකාරයට නොවරදවා සහභාගීවීමේ වැදගත්කම ගැන වෛද්‍ය වෘත්තිකයෙකු (ඔබේ වෛද්‍යවරයා, හෙදිය, පෝෂණවේදියා හෝ වෙනත් වෛද්‍ය කාර්ය මණ්ඩල නිලධාරියෙකු) ඔබ සමඟ අවසන්වරට කතා කළේ කවදාද?		☐ මේ සතියේ(1)<br>☐ පසුගිය සතියේ(2)<br>☐ මාසයකට පෙර(3)<br>☐ මාස කීපයකට පෙර (4)<br>☐ මම මුලින්ම රුධිර කාන්දුකරණ (ලේ පිරිසිදු කිරීමේ) ප්‍රතිකාරය ආරම්භ කල දිනයේ (5)<br>☐ කවදාවත් නැත/ මතක නැත .(6)<br>☐ වෙනත් (සඳහන් කරන්න.)(7)
10.	රුධිර කාන්දුකරණ (ලේ පිරිසිදු කිරීමේ) ප්‍රතිකාරයේදී නියමිත සම්පූර්ණ වේලාව රැඳී සිටීමේ වැදගත්කම ගැන වෛද්‍ය		☐ සෑම රුධිර කාන්දුකරණ (ලේ පිරිසිදු කිරීමේ) ප්‍රතිකාර දිනයකදීම.(1)<br>☐ සෑම සතියකදීම.(2)

	වෘත්තිකයෙකු (ඔබේ වෛද්‍යවරයා, හෙදිය, පෝෂණවේදියා හෝ වෙනත් වෛද්‍ය කාර්ය මණ්ඩල නිලධාරියෙකු) ඔබව කොපමණ කාලයකට වරක් දැනුවත් කරන්නේද?		☐ සෑම මසකදීම.(3)<br>☐ මාගේ රුධිර හෝ වෙනත් පරීක්ෂණ වාර්තාවක් අසාමාන්‍ය වූ විට .(4)<br>☐ කලාතුරකිනි.(5)<br>☐ කවදාවත් නැත/ මතක නැත .(6)<br>☐ වෙනත් (සඳහන් කරන්න.)(7)
11.	ඔබේ රුධිර කාන්දුකරණ (ලේ පිරිසිදු කිරීමේ) ප්‍රතිකාර කාලසටහන නිවැරදිව අනුගමනය කිරීම කොතරම් වැදගත්දැයි ඔබ සිතන්නේද?		☐ අතිශයින්ම වැදගත්(1)<br>☐ ඉතාමත් වැදගත්ය.(2)<br>☐ මධ්‍යස්ථව වැදගත් වේ.(3)<br>☐ ඉතා සුළු වශයෙන් වැදගත් වේ.(4)<br>☐ වැදගත් නොවේ.(5)
12.	ඔබේ රුධිර කාන්දුකරණ (ලේ පිරිසිදු කිරීමේ) ප්‍රතිකාර ලබාගැනීමේ කාලසටහන නිවැරදිව අනුගමනය කිරීම වැදගත් වන්නේ ඇයි? (වඩාත් ගැළපෙන පිළිතුර තෝරන්න.)		☐ මාගේ වකුගඩු රෝගය සඳහා මෙම ප්‍රතිකාර ක්‍රමය නියමිත පරිදි ලබාගැනීම ඉතා වැදගත් යැයි මා අවබෝධ කරගෙන ඇති නිසා.(1)<br>☐ රුධිර කාන්දුකරණ (ලේ පිරිසිදු කිරීමේ) ප්‍රතිකාරය නියමිත ලෙස පවත්වාගෙන යාම මාගේ ශරීරය නිරෝගීව තැබීමට හේතු වන නිසා(2)<br>☐ වෛද්‍ය වෘත්තිකයෙකු (වෛද්‍යවරයා, හෙදිය, පෝෂණවේදනිය) පැවසූ නිසා(3)<br>☐ රුධිර කාන්දුකරණ (ලේ පිරිසිදු කිරීමේ) ප්‍රතිකාරය අතපසු වීම නිසා රෝගීවීමේ අත්දැකීම මා ලැබූ නිසා/ රෝහල්ගතවීමේ අත්දැකීම මා ලැබූ නිසා(4)<br>☐ රුධිර කාන්දුකරණ (ලේ පිරිසිදු කිරීමේ) ප්‍රතිකාර නියමිත කාලසටහනකට අනුව සිදුකිරීම වැදගත් යැයි නොහැඟේ.(5)<br>☐ වෙනත් (සඳහන් කරන්න)................ (6)
13.	වෛද්‍යවරයා විසින් නිර්දේශ කරන ලද රුධිර කාන්දුකරණ (ලේ පිරිසිදු කිරීමේ) ප්‍රතිකාර සිදුකිරීමේ කාලසීමාවේදී ඔබ කොතරම් අපහසුතාවන් වලට ලක්වූයේද?		☐ අපහසුතාවයන් නොමැත.(1)<br>☐ ඉතා කුඩා අපහසුතාවයන් ඇත.(2)<br>☐ මධ්‍යස්ථ අපහසුතාවයන් ඇත.(3)<br>☐ ඉතා විශාල ප්‍රමාණයෙන් අපහසුතාවයන් ඇත.(4)<br>☐ දරාගැනීමට නොහැකි තරමේ අපහසුතාවයන් ඇත.(5)

14.	පසුගිය මාසයේදී ඔබට කොපමණ රුධිර කාන්දුකරණ (ලේ පිරිසිදු කිරීමේ) සම්පූර්ණ ප්‍රතිකාර වාර ප්‍රමාණයක් මගහැරී ඇත්ද?		☐ 0 (1)<br>☐ 1 (2)<br>☐ 2 (3)<br>☐ 3 (4)<br>☐ > 4 (5)
15.	පසුගිය මාසයේදී, ඔබේ රුධිර කාන්දුකරණ (ලේ පිරිසිදු කිරීමේ) ප්‍රතිකාරය මගහැරීයාමේ ප්‍රධාන හේතුව කුමක්ද?		☐ අදාළ නැත. (මා හට කිසිදු ප්‍රතිකාර වාරයක්වත් මග හැරී නැත.)(1)<br>☐ ප්‍රවාහන පහසුකම් නැතිවීම.(2)<br>☐ මා හට වෙනත් කාර්යයක් සිදුකිරීමට තිබීම නිසා(3)<br>☐ රුධිර කාන්දුකරණ (ලේ පිරිසිදු කිරීමේ) ප්රවේශයේ රුධිරකැටි ඇතිවීම (කැතිටර්, ෆිස්ටියුලා, ග්රාෆ්ට්.).(4)<br>☐ වෛද්‍යවරයෙකුගේ (ශෛල්‍ය වෛද්‍ය/වෛද්‍ය) හමුවීමක් නිසාය. (5)<br>☐ මා රෝහල් ගත කළ නිසා(7)/මා හදිසි ප්‍රතිකාර අංශයට ඇතුළත් කළ නිසා(6)<br>☐ අමතකවීම නිසා(7)<br>☐ ආර් ථික අපහසුතාවයක් නිසා (8)<br>☐ "යාමට අවශ්‍යතාවයක් නැතිවීම"/"යාමට නොහැකි විය." (16වෙනි ප්‍රශ්ණය වෙත යන්න.)(9)<br>☐ වෙනත් (කරුණාකර සඳහන් කරන්න)..........(10)
16.	(ඉහත උත්තර ඇසුරින් "යාමට අවශ්‍යතාවයක් නැත"/"යාමට නොහැකි විය" යන උත්තරය තෝරාගත් අය පමණක් මෙම ප්‍රශ්නයට පිළිතුරු සපයන්න.)<br>ඔබට රුධිර කාන්දුකරණ (ලේ පිරිසිදු කිරීමේ) මධ්‍යස්ථානය වෙත යාමට අවශ්‍ය නොවූයේ ඇයි?		☐ රුධිර කාන්දුකරණ (ලේ පිරිසිදු කිරීමේ) ක්‍රියාවලිය මා ඉක්මන් කලබල වන තත්වයකට හෝ බියවන තත්වයකට පත් කරන නිසා (1)<br>☐ මට වමනය යාම/බඩඑළිය යාමේ රෝගී තත්වයක් ඇති වූ නිසා(2)<br>☐ මාංශ පේශි පෙරලීමක් සිදුවූ නිසා (3)<br>☐ ප්‍රතිකාර අතරතුරදී මට නිතර කුසගිනි සෑදෙන නිසා(4)<br>☐ මාගේ ශාරීරික අපහසුතාවයක් නිසා (5)<br>☐ වෙනත් හේතුවක් නිසාවෙන් මා රෝගී වූ නිසා (පැහැදිළි කරන්න.)(6)<br>☐ මා මානසික අවපීඩනයෙන් සිටි නිසා(7)<br>☐ වෙනත්(8)........................

17.	පසුගිය මාසයේදී කොපමණ වාරයක් ඔබ ඔබේ රුධිර කාන්දුකරණ (ලේ පිරිසිදු කිරීමේ) ප්‍රතිකාර කාලය කෙටි කළාද?		☐ කිසිදු අවස්ථාවක ප්‍රතිකාර වාර කෙටි කර නැත (1)<br>☐ එක්වරක්(2)<br>☐ දෙවරක්(3)<br>☐ තුන්වරක්(4)<br>☐ හතර හෝ පස් වරක්(5)<br>☐ වෙනත්(වාර ගණන සඳහන් කරන්න)............(6)
18.	පසුගිය මාසයේදී, ඔබගේ රුධිර කාන්දුකරණ (ලේ පිරිසිදු කිරීමේ) ප්‍රතිකාර කාලය කෙටි කළා නම්, සාමාන්‍යයෙන් මිනිත්තු ගණන කොපමණද?		☐ අදාළ නැත. (මා කිසිදු අවස්ථාවක ප්‍රතිකාර වාර කෙටි කර නැත.)(1)<br>☐ විනාඩි 15(2)<br>☐ විනාඩි 30(3)<br>☐ පැය 01(4)<br>☐ පැය 01 ට වැඩි(5)<br>☐ වෙනත් (සඳහන් කරන්න.)(6)<br>පසුගිය මාසයේ ප්‍රතිකාර කෙටි කළ වාර ගණන එකකට වඩා වැඩිනම් ප්‍රතිකාර කෙටි කළ වේලාවන් සඳහන් කිරීමට මෙම තීරුව භාවිතා කරන්න.)..
19.	ඔබගේ රුධිර කාන්දුකරණ (ලේ පිරිසිදු කිරීමේ) ප්‍රතිකාර කාලය කෙටි කර ගැනීමට ප්‍රධාන හේතුව කුමක්ද?		☐ අදාළ නැත. (මා කිසිදු අවස්ථාවක ප්‍රතිකාර වාර කෙටි කර නැත.)(1)<br>☐ මාංශ පේශී පෙරළීමක් සිදුවූ නිසා (2)<br>☐ වැසිකිළිය පාවිච්චි කිරීමට සිදුවීම.(3)<br>☐ නොසන්සුන්තාවයක් නිසාය.(4)<br>☐ රුධිර පීඩනය අඩුවූ නිසාය.(5)<br>☐ ප්‍රවේශයේ රුධිරකැටි ඇතිවීම (කැතිටර්, ෆිස්ටියුලා, ග්‍රාෆ්ට්.)(6)<br>☐ වෛද්‍යවරයෙකුගේ (ශෛල්‍ය/වෛද්‍ය) හමුවීමක් තිබූ නිසාය.(7)<br>☐ පෞද්ගලික කටයුත්තක්/හදිසි අවශ්‍යතාවයක් නිසාය.(8)<br>☐ රාජකාරී / වැඩ කටයුතු නිසාය.(9)<br>☐ ප්‍රවාහන පහසුකම්වල ප්‍රශ්න නිසාය.(10)<br>☐ කාර්යය මණ්ඩලයේ තීරණයක් නිසා (ඒ ඇයිදැයි කරුණාකර පැහැදිලි කරන්න. උදා:- දුර්වල රුධිර ගමනාගමනය, රුධිරයට

			සම්බන්ධ කර ඇති උපාංගවල දෝශයක් යන දේවල්ය.)(11)<br>☐ රුදී සිටීමට අකමැති වීම (12)<br>☐ වෙනත් (කරුණාකර සඳහන් කරන්න)…………(13)
**III.**	**<u>ඖෂධ</u>**		
20.	ඔබගේ ඖෂධ ගැන වෛද්‍ය වෘත්තිකයෙකු(ඔබේ වෛද්‍යවරයා, හෙදිය, පෝෂණවේදියා හෝ වෙනත් වෛද්‍ය කාර්ය මණ්ඩල නිලධාරියෙකු) ඔබ සමඟ අවසාන වරට කතා කළේ කවදාද?		☐ මේ සතියේ(1)<br>☐ පසුගිය සතියේ(2)<br>☐ මාසයකට පෙර(3)<br>☐ මාස කීපයකට පෙර (4)<br>☐ මම මුලින්ම රුධිර කාන්දුකරණ (ලේ පිරිසිදු කිරීමේ) ප්‍රතිකාරය ආරම්භකල දිනයේ (5)<br>☐ කවදාවත් නැත/ මතක නැත .(6)<br>☐ වෙනත් (සඳහන් කරන්න.)(7)
21.	ඔබට නියම කළ ඖෂධ නිර්දේශ කල පරිදි ගැනීමේ වැදගත්කම ගැන වෛද්‍ය වෘත්තිකයෙකු (ඔබේ වෛද්‍යවරයා, හෙදිය, පෝෂණවේදියා හෝ වෙනත් වෛද්‍ය කාර්ය මණ්ඩල නිලධාරියෙකු) ඔබ සමඟ කොපමණ කාලයකට වරක් කතා කරන්නේද?		☐ සෑම රුධිර කාන්දුකරණ (ලේ පිරිසිදු කිරීමේ) ප්‍රතිකාරයකදීම. (1)<br>☐ සෑම සතියකදීම.(2)<br>☐ සෑම මසකදීම.(3)<br>☐ මාගේ රුධිර හෝ වෙනත් පරීක්ෂණ වාර්තාවක් අසාමාන්‍ය වූ විට (උදා: අධිරුධිර පීඩනය)(4)<br>☐ කලාතුරකිනි.(5)<br>☐ කවදාවත් නැත/ මතක නැත (6)<br>☐ වෙනත් (සඳහන් කරන්න.)(7)
22.	ඔබ සිතන ආකාරයට ඔබගේ ඖෂධ නිර්දේශ කල පරිදි ගැනීම කොතරම්දුරට වැදගත්ද?		☐ අතිශයින්ම වැදගත්(1)<br>☐ ඉතාමත් වැදගත්ය.(2)<br>☐ මධ්‍යස්ථව වැදගත් වේ.(3)<br>☐ ඉතා සුළු වශයෙන් වැදගත් වේ.(4)<br>☐ වැදගත් නොවේ.(5)
23.	ඔබ සිතන ආකාරයට ඖෂධ නිර්දේශ කල පරිදි ගැනීම වැදගත්වන්නේ ඇයි?<br>(වඩාත් ගැළපෙන පිළිතුර තෝරන්න.)		☐ සම්පූර්ණයෙන්ම මගේ වකුගඩුවල තත්වය තේරුම් ගත්ත නිසා, නියම කර ඇති පරිදි ඖෂධ ලබා ගැනීම අවශ්‍යයි (1)<br>☐ මගේ සිරුර සෞඛ්‍ය සම්පන්නව තබා ගැනීමට ඖෂධ ගැනීම වැදගත්ය.(2)<br>☐ වෛද්‍ය වෘත්තිකයෙකු (මගේ වෛද්‍යවරයා, හෙදි නිලධාරිනිය, පෝෂණවේදිනිය හෝ

			වෙනත් වෛද්‍ය නිලධාරියෙක්) එය කිරීමට මට පැවසූ නිසා(3)<br>☐ මම ඖෂධ ගැනීම අතපසු කළ විට අසනීප වීමේ අත්දැකීම මට ලැබුණු නිසා/අසනීපවී රෝහල්ගත වීමේ අත්දැකීම මට ලැබුණු නිසා .(4)<br>☐ මම ඖෂධ ලබාගැනීම ඉතා වැදගත් කියා සිතන්නේ නැහැ.(5)<br>☐ වෙනත් (සඳහන් කරන්න.)(6)...........................
24.	නිර්දේශිත ඖෂධ ලබාගැනීමේදී ඔබට අපහසුතාවයන් ඇතිවී තිබේද?		☐ නැත.(1)<br>☐ ඔව්.(2)
25.	නිර්දේශිත ඖෂධ ලබාගැනීමේදී ඔබට කොපමණ අපහසුතාවයන් ඇතිවී තිබේද?		☐ අපහසු නැත.(1)<br>☐ ටිකක් අපහසුයි.(2)<br>☐ මධ්‍යස්ථව අපහසුයි.(3)<br>☐ ගොඩක් අපහසුයි.(4)<br>☐ අතිශයින්ම අපහසුයි.(5)
26.	ඔබට නියමිත ඖෂධ, ඔබ පසුගිය සතියේ කොපමණ වාරයක් අතපසු කර ඇත්ද?		☐ කිසිම අවස්ථාවක නැත (1)<br>☐ ඉතා කලාතුරකිනි.(2)<br>☐ සතියෙන් බාගයක් පමණ.(3)<br>☐ ගොඩක් වේලාවට.(4)<br>☐ හැමවිටම.(5)
27.	පසුගිය සතියේ ඔබට නියමිත ඖෂධ ගැනීම අතපසු වීමට ප්‍රධාන හේතුව කුමක්ද?		☐ අදාළ නොවේ: මම මගේ ඖෂධ අතපසු කලේ නැත.(1)<br>☐ ඖෂධ ලබාගැනීමට අමතක වීම (2)<br>☐ ඖෂධ ඇණවුම් කිරීමට අමතක වීම (3)<br>☐ ඖෂධ මිල අධික නිසා (4)<br>☐ මම රෝහල්ගත වූ නිසා (5)<br>☐ මගේ සහයට කිසිවකු නොමැති වීම නිසා(6).<br>☐ අතුරු ආබාධ ඇති වූ නිසා (7) (28වෙනි ප්‍රශ්නයට යන්න.)<br>☐ වෙනත්.(8)...
28.	ඉහත ප්‍රශ්නය සඳහා පිළිතුර "අතුරු ආබාධ" නම්, ඖෂධය (ඖෂධ) ලබාගැනීමේදී ඔබට කුමන ආකාරයේ අතුරු ආබාධ ඇතිවුයේද? (පිළිතුරුවලින් වඩාත් ගැළපෙන පිළිතුරු තෝරන්න.)		☐ ආහාර රුචිය නැතිවීම.(1)<br>☐ ඕක්කාරය/වමනය යාම/පාචනය/ මල බද්ධය(2)<br>☐ බඩේ අමාරුව (කැක්කුම)(3)<br>☐ කරකැවිල්ල (4)

			☐ ඔළුව කැක්කුම.(5)<br>☐ කැසීම/සමේ ගැටලු.(6)<br>☐ වෙනත් (රෝග ලක්ෂණ දක්වන්න.)(7)
**IV.**	**දියර පාලනය**		
29.	වෛද්‍ය වෘත්තිකයෙකු විසින් (ඔබේ වෛද්‍යවරයා, හෙදිය, පෝෂණවේදියා හෝ වෙනත් වෛද්‍ය කාර්ය මණ්ඩල නිලධාරියෙකු) ඔබට ලබාගත හැකි දියර සීමාවන් පිළිබඳ අවසාන වරට සාකච්ඡා කලේ කවදාද?		☐ මේ සතියේ(1)<br>☐ ගිය සතියේ(2)<br>☐ මීට මාසයකට පමණ පෙර(3)<br>☐ මාස කීපයකට පෙර(4)<br>☐ මම මුලින්ම රුධිර කාන්දුකරණ (ලේ පිරිසිදු කිරීමේ) ප්‍රතිකාරය ආරම්භකල දිනයේ (5)<br>☐ කවදාවත් නැත/ මතක නැත (6)<br>☐ වෙනත් (සඳහන් කරන්න.)(7)
30.	වෛද්‍ය වෘත්තිකයෙකු විසින් (වෛද්‍යවරයා, හෙදිය, පෝෂණවේදියා හෝ වෙනත් වෛද්‍ය කාර්ය මණ්ඩල නිලධාරියෙකු) ඔබ ගන්නා දියර සීමාකිරීමේ වැදගත්කම පිළිබඳ කොපමණ වාර ගණනක් ඔබ සමඟ කතා කරනවාද ?		☐ සෑම රුධිර කාන්දුකරණ (ලේ පිරිසිදු කිරීමේ) ප්‍රතිකාරයකදීම(1)<br>☐ සෑම සතියකදීම(2)<br>☐ සෑම මසකදීම(3)<br>☐ මාගේ රුධිර හෝ වෙනත් පරීක්ෂණ වාර්තාවක් අසාමාන්‍ය වූ විට(4)<br>☐ කලාතුරකින්(5)<br>☐ කවදාවත් නැත/ මතක නැත (6)<br>☐ වෙනත් (සඳහන් කරන්න)(7)............
31.	ඔබ පසුගිය සතිය තුළ කොපමණ වාර ගණනක් නිර්දේශ කළ දියර සීමා කිරීමේ ක්‍රියාවලිය අනුගමනය කළාද?		☐ සෑම විටම (1)<br>☐ බොහෝ විට(2)<br>☐ භාගයක් දුරට(3)<br>☐ ඉතා කලාතුරකින් (4)<br>☐ කවදාවත් නැත/ මතක නැත .(5)<br>☐ වෙනත් (සඳහන් කරන්න)(7)............
32.	ඔබ සිතන ආකාරයට දියර සීමා කිරීම කොපමණ වැදගත්ද?		☐ අතිශයින්ම වැදගත්(1)<br>☐ ඉතා වැදගත්(2)<br>☐ මධ්‍යස්ථව වැදගත්.(3)<br>☐ සුළුවෙන් වැදගත්(4)<br>☐ වැදගත් නැත.(5)
33.	ඔබගේ දියර පානය සීමා කිරීම වැදගත් වනවා කියා ඔබ සිතන්නේ ඇයි?<br>(වඩාත් ගැළපෙන පිළිතුර තෝරන්න.)		☐ මාගේ වකුගඩු ආබාධය සඳහා මා ගන්නා දියර සීමා කිරීම ඉතා වැදගත් බව මා අවබෝධ කරගෙන සිටින නිසාය.(1)

			□ දියර ලබාගැනීම සීමා කිරීම මාගේ ශරීරය සෞඛ්‍යමත්ව තබන නිසාය.(2)<br>□ වෛද්‍ය වෘත්තිකයෙකු (වෛද්‍යවරයා, හෙදිය, පෝෂණවේදියා වෙනත් වෛද්‍ය කාර්ය මණ්ඩල නිලධාරියෙකු) මට එසේ කිරීමට පැවසූ නිසා(3)<br>□ මා වැඩිපුර දියර පානය කල විට අසනීප වූ නිසා/ විශාල වශයෙන් දියර පානය කිරීම නිසා මා හට රෝහල්ගත වීමට සිදුවීම (4)<br>□ මම දියර සීමා කිරීම ඉතා වැදගත් කියා සිතන්නේ නැහැ (5)<br>□ වෙනත් (සඳහන් කරන්න)(6)................
34.	නිර්දේශිත දියර සීමා කිරීම අනුගමනය කිරීමේදී ඔබට අසීරුතා ඇති වුයේද?		□ නැත.(1)<br>□ ඔව්.(2)
35.	නිර්දේශිත දියර සීමා කිරීම අනුගමනය කිරීමේදී ඔබට කොපමණ අසීරුතා ඇති වුයේද?		□ අසීරුතාවයක් නැත.(1)<br>□ ඉතා අඩුවෙන් අසිරුතාවයන් ඇත.(2)<br>□ සාමාන්‍ය/මධ්‍යස්ථ අසීරුතාවයන් ඇත.(3)<br>□ විශාල වශයෙන් අසීරුතා ඇත.(4)<br>□ මට කිසිම විටක නිර්දේශ කල පරිදි දියර පාලනය සිදු කිරීමට නොහැකි විය(5)
36.	නිර්දේශිත දියර සීමාකිරීම අනුගමනය කිරීම සම්බන්ධයෙන් අපහසුතා ඇත්නම්, ඒවා කුමන ආකාරයේ අපහසුතාවයන්ද?		□ දියර පාලනයට කැමැත්තක් නැත.(1)<br>□ මට මගේ දියර පාලනය කිරීමට නොහැකි වීම (2)<br>□ දියර සීමා කිරීම සම්බන්ධයෙන් අවබෝධයක් නොමැතිවීම .(3)<br>□ දියර පාලනය කිරීමේදී ක්ලාන්ත ගතියක් ඇතිවේ(4).<br>□ දියර පානය නොකර ආහාර ගැනීමට නොහැක(5).<br>□ වෙනත්(6)....................
37.	පසුගිය සතිය තුළ කොපමණ වාර ගණනක් නිවසේදී ඔබ ඔබගේ බර කිරා බැලුවේද? (රුධිර කාන්දුකරණ මධ්‍යස්ථානයෙන් පිටත)		□ තුන්වරකට වඩා වැඩියි.(1)<br>□ තුන්වරක්ය.(2)<br>□ දෙවරක්ය.(3)<br>□ එක්වරක්ය.(4)<br>□ කවදාවත් නැත.(5)<br>□ වෙනත්(6)................

38.	දෛනිකව ඔබගේ බර කිරා බැලීම කොපමණ වැදගත් වේදැයි ඔබ සිතන්නේද?		☐ අතිශයින්ම වැදගත්(1)<br>☐ ඉතාමත් වැදගත්ය.(2)<br>☐ මධ්‍යස්ථව වැදගත් වේ.(3)<br>☐ ඉතා සුළු වශයෙන් වැදගත් වේ.(4)<br>☐ වැදගත් නොවේ.(5)
**V.**	**ආහාර වේල**		
39.	වෛද්‍ය වෘත්තිකයෙකු විසින් (ඔබේ වෛද්‍යවරයා, හෙදිය, පෝෂණවේදියා හෝ වෙනත් වෛද්‍ය කාර්ය මණ්ඩල නිලධාරියෙකු) අවසාන වරට ඔබගේ ආහාරවේල සම්බන්ධව සාකච්ඡා කළේ කවදාද?		☐ මෙම සතියේදී(1)<br>☐ ගිය සතියේදී(2)<br>☐ මසකට පෙර(3)<br>☐ මාස කීපයකට පෙර (4)<br>☐ මා මුලින්ම රුධිර කාන්දුකරණ (ලේ පිරිසිදු කිරීමේ) ප්‍රතිකාර ආරම්භ කල දිනදී(5)<br>☐ කවදාවත් නැත/ මතක නැත .(6)<br>☐ වෙනත් (පැහැදිලි කරන්න)(7)...............
40.	වෛද්‍ය වෘත්තිකයෙකු විසින් (වෛද්‍යවරයා, හෙදිය, පෝෂණවේදියා හෝ වෙනත් වෛද්‍ය කාර්ය මණ්ඩල නිලධාරියෙකු) නිවැරදි ආහාර රටාවක් අනුගමනය කිරීමේ වැදගත්කම පිළිබඳ කොපමණ කාලයකට වරක් ඔබ සමඟ සාකච්ඡා කරන්නේද?		☐ සෑම ප්‍රතිකාර අවස්ථාවකදීම(1)<br>☐ සෑම සතියකදීම(2)<br>☐ සෑම මසකදීම(3)<br>☐ මාගේ රුධිර හෝ වෙනත් පරීක්ෂණ වාර්තාවක් අසාමාන්‍ය වූ විට (4)<br>☐ කලාතුරකින්(5)<br>☐ කවදාවත් නැත/ මතක නැත (6)<br>☐ වෙනත් (පැහැදිලි කරන්න)(7)...........
41.	ඔබ දිනපතා ගන්නා ආහාර පිළිබඳ සැලකිලිමත් වීම කෙතරම් වැදගත්දැයි ඔබ සිතන්නේද?		☐ අතිශයින්ම වැදගත්(1)<br>☐ ඉතාමත් වැදගත්ය.(2)<br>☐ මධ්‍යස්ථව වැදගත් වේ.(3)<br>☐ ඉතා සුළු වශයෙන් වැදගත් වේ.(4)<br>☐ වැදගත් නොවේ.(5)
42.	ඔබ සිතන ආකාරයට දිනපතා ඔබ ගන්නා ආහාර පිළිබඳව විමසිලිමත් වීම කොතරම් වැදගත්ද?(වඩාත් ගැළපෙන පිළිතුර තෝරන්න.)		☐ මාගේ වකුගඩු ආබාධය සඳහා මාගේ ආහාර වේල් පිළිබඳ විමසිලිමත්වීම වැදගත් බව මා අවබෝධ කරගෙන ඇති බැවිනි.(1)<br>☐ මාගේ ආහාර වේල් නිසා මාගේ ශරීරය සෞඛ්‍යමත්ව තිබෙන නිසාය.(2)<br>☐ වෛද්‍ය වෘත්තිකයෙකු (වෛද්‍යවරයා, හෙදිය, පෝෂණවේදියා වෙනත්) මට එසේ කිරීමට පැවසූ නිසාය.(3)

			☐ මා මාහට නුසුදුසු ආහාර ගත්විට අසනීප වූ නිසා හෝ රෝහල්ගත වීමට සිදුවූ නිසා (4)<br>☐ මම මාගේ ආහාර වේල් ගැන සැලකිලිමත්වීම වැදගත් කියා සිතන්නේ නැහැ (5)<br>☐ වෙනත් (පැහැදිළි කරන්න)(6)......................
43.	නිර්දේශ කරන ලද ආහාර රටාව අනුගමනය කිරීමේදි ඔබට අසීරුතා ඇතිවූයේද?		☐ නැත.(1)<br>☐ ඔව්.(2)
44.	නිර්දේශ කරන ලද ආහාර රටාව අනුගමනය කිරීමේදි ඔබට කොතරම් අසීරුතා ඇතිවූයේද?		☐ අසීරුතා නැත.(1)<br>☐ සුළු අසීරුතා ඇත.(2)<br>☐ මධ්‍යස්ථව අසීරුතා ඇත.(3)<br>☐ විශාල වශයෙන් අසීරුතා ඇත.(4)<br>☐ අනුමත කර ඇති ආහාර වේල් රටාව අනුගමනය කිරීමට කිසිම විටක නොහැකි විය.(5)
45.	නිර්දේශ කරන ලද ආහාර රටාව අනුගමනය කිරීමේදී ඔබට කුමන ආකාරයේ අපහසුතා ඇතිවේද?		☐ අදාළ නැත. (අසීරුතා නැත)(1)<br>☐ මාහට ආහාර රුචියක් නොමැතිවීම(2)<br>☐ කෑමට අවශ්‍ය දේ පාලනය කිරීමට මා අකමැතිය.(3)<br>☐ මට කෑමට නිර්දේශ නොකළ සමහර ආහාර ගැනීමෙන් වැළකී සිටිය නොහැකි විය(4)<br>☐ කුමන ආකාරයේ ආහාර වේලක් ලබා ගත යුතුදැයි මට අවබෝධයක් නොමැත.(5)<br>☐ වෙනත් (පැහැදිළි කරන්න)(6)................
46.	පසුගිය සතිය තුළ කොපමණ වාර ගණනක් ඔබට නිර්දේශ කරන ලද ආහාර රටාව අනුගමනය කළාද?		☐ සෑම වේලාවකදීම.(1)<br>☐ බොහෝ වෙලාවට (2)<br>☐ භාගයක් දුරට (3)<br>☐ ඉතා අඩුවෙනි.(4)<br>☐ කිසිම විටක නැත.(5)
