# Supplementary material for: Psychometric properties of End Stage Renal Disease-Adherence Questionnaire-Sinhalese version among patients receiving haemodialysis
Source: PLoS One. 2023 Oct 20;18(10):e0292938. doi: 10.1371/journal.pone.0292938 (PMC10588851; doi:10.1371/journal.pone.0292938)
Supplement: S2 File — (PDF) [file pone.0292938.s002.pdf]

S2 File: Cultural adaptation of SINESRD-AQ

Modifications to the answers in the translated Sinhala version of ESRD-AQ as suggested by panel of experts

Item No.	Item	Common forward translated Sinhala version of ESRD-AQ. (answer identical to original English version)	Final Sinhala version of ESRD-AQ after face and content validation (This English version of answer is identical to the final Sinhala version of the answer)
01	When did you start or restart your haemodialysis treatment?	Beginning date – Month/year …………<br>Restarting date if you restarted hemodialysis<br>Month/year …………	Month and year of beginning/ restarting the haemodialysis treatment<br><br>Month/year …………
02	Have you ever undergone chronic peritoneal dialysis?	No<br>Yes<br>I had peritoneal dialysis from ……………………………….<br>Month/year to Month/year	No<br>Yes<br>If yes,<br>Month/year …to Month/year ………
03	Have you had a kidney transplant?	No<br>Yes<br>I had a kidney transplant once from ……………to ………………….<br>Month/Year Month/Year<br><br>Or<br>I had kidney transplant twice from ……………to ………………….<br>Month/Year Month/Year<br><br>……………to ………………….<br>Month/Year Month/Year<br>And from<br>…………to………………<br>Month/Year Month/Year	No<br><br>Yes<br><br>If yes,<br>How many times?<br>…………………….
04	What type of transportation do you use to go to the dialysis center?	1. Personal transportation<br>2. Bus<br>3. Taxi<br>4. Medical transportation van<br>5. Other (please specify)………	1. Personal transportation<br>2. Bus<br>3. Taxi<br>4. Ambulance/ medical transportation<br>5. Other (please specify) ………
06	How many days a week do you receive hemodialysis treatment?	1. 2 days or less<br>2. 3days<br>3. 4 days<br>4. More than 4 days<br>5. More than 5 days	1. Only 1 day<br>2. 2 days<br>3. 3days<br>4. 4 days<br>5. More than 4 days

07	How many hours are you treated for each hemodialysis?	1. Less than 3 hours<br>2. 3 hours<br>3. 3 hours and 15 minutes<br>4. 3 hours and 30 minutes<br>5. 3 hours and 45 minutes<br>6. 4 hours<br>7. More than 4 hours<br>8. Other (please specify the hours)……………….	1. Less than 3 hours<br>2. 3 hours<br>3. 3 hours and 30 minutes<br>4. 4 hours<br>5. More than 4 hours<br>6. Other (please specify the hours)……………….
08	Is your dialysis schedule convenient for you?	1. Yes<br>2. No, because I have to come to the dialysis center too early in the day<br>3. No, because I have to come to the dialysis center too late in the day<br>4. No, because of my work schedule<br>5. No, because it falls on my mealtime and I am hungry during the dialysis treatment<br>6. No, because it falls on my medication time and I have to take medicine/insulin<br>7. No, because of (other)………..	1.Yes<br>2.No, because I have to come to the dialysis center too early in the day<br>3. No, it is not aligned with my work schedule.<br>4. No, I have to come for hemodialysis at given time from the hospital.<br>5. No, it takes much time to me to reach the hospital.<br>6. Other……………..
09,20,29, 39	When was the last time a medical professional (doctor, nurse, dietician or any other medical staff) talked to you about,<br><br>09. the importance of not missing your dialysis treatment?<br>20. your medications?<br>29. about your fluid restrictions?<br>39. your meal plan?	1. This week<br>2. Last week<br>3. One month ago<br>4. More than a month ago<br>5. When I first began dialysis treatment<br>6. Never<br>7. Other (please specify) …………………	1. This week<br>2. Last week<br>3. One month ago<br>4. Few months ago<br>5. When I first began dialysis treatment<br>6. Never/can't remember<br>7. Other (please specify) …
10, 21, 30, 40	How often does a medical professional (doctor, nurse,	1. Every dialysis treatment<br>2. Every week<br>3. Every month<br>4. Every 2 to 3 months	1. Every dialysis treatment<br>2. Every week<br>3. Every month

	dietician or any other medical staff) talked to you about<br>10.the importance of completing the entire dialysis session?<br><br>21. the importance of taking medications as prescribed?<br><br>30. the importance of fluid restriction?<br><br>40. the importance of following a proper meal plan?	5. Every 4 to 6 months<br>6. When blood or other test results (for example, blood pressure) are abnormal<br>7. Rarely<br>8. Irregularly<br>9. Never<br>10. Other (please specify):	4. When blood or other test results (for example, blood pressure) are abnormal<br>5. Rarely<br>6. Never/can't remember<br>7. Other (please specify):
14	During the last month, how many dialysis treatments did you miss completely?	1. Missed one dialysis treatment<br>2. Missed two dialysis treatments<br>3. Missed three dialysis treatments<br>4. Missed four or more dialysis treatments<br>5. None (I did not miss any treatments)	1. 0<br>2. 1<br>3. 2<br>4. 3<br>5. >4
15	What was the main reason for missing your dialysis treatment during last month?	1. Not applicable. (I did not miss any treatment)<br>2. Transportation problems<br>3. I had other things/work to do (Please explain)<br>4. Clotting of the hemodialysis access (catheters, graft or fistula)<br>5. Attend a medial (physician or surgical) appointment<br>6. I was admitted to the emergency treatment unit<br>7. I was hospitalized<br>8. Forgot<br>9. "Didn't want to go" or "Couldn't go" (Go to the next question: Question 16)<br>10. Other (Please specify): ………	1. Not applicable. (I did not miss any treatment)<br>2. Transportation problems<br>3. I had other things/work to do (Please explain)<br>4. Clotting of the hemodialysis access (catheters, graft or fistula)<br>5. Attending a medial or surgical appointment<br>6. I was hospitalized/ admitted to the emergency treatment unit<br>7. Forgot<br>8. Due to financial issues<br>9. "Didn't want to go" or "Couldn't go" (Go to the next question: Question 16)<br>10. Other (Please specify): …

18	During the last month, if your dialysis treatment duration was shortened, what was the average duration in minutes?	1. Not applicable (my dialysis treatment duration was never shortened)<br>2. Less than 10 minutes or 10 minutes<br>3. 11 to 20 minutes<br>4. 21 to 30 minutes<br>5. More than 31 minutes<br>6. Other (please specify)	1. Not applicable (my dialysis treatment duration was never shortened)<br>2. 15 minutes<br>3. 30 minutes<br>4. 01 hour<br>5. More than 01 hour<br>6. Other (please specify)
11, 22, 32, 38, 41	11. How important do you think it is necessary to follow hemodialysis schedule?<br><br>22. How important do you think it is necessary to take your medications as scheduled?<br><br>32. How important do you think it is necessary to limit fluid intake?<br><br>38. How important do you think it is necessary to weigh yourself daily?<br><br>41. How important do you think it is necessary to watch/be careful about the types of food you eat each day?	1. Highly important<br>2. Very important<br>3. Moderately important<br>4. A little important<br>5. Not important	1. Extremely important<br>2. Very important<br>3. Moderately important<br>4. A little important<br>5. Not important
12	Why do you think it is important to follow your dialysis schedule? (please choose one best answer that applies to you)	1. Because I fully understand that my kidney condition requires dialysis as scheduled<br>2. Because following the dialysis schedule is important to keep my body healthy	1. Because I fully understand that my kidney condition requires dialysis as scheduled<br>2. Because following the dialysis schedule is important to keep my body healthy

		3. Because medical professional (my doctor, nurse or dietitian) told me to do so<br>4. Because I had an experience that I was sick after I missed dialysis<br>5. Because I had an experience that I was hospitalized after I missed dialysis<br>6. I don't think following the dialysis schedule is very important to me<br>7. Other (Specify) …………	3. Because medical professional (my doctor, nurse or dietitian) told me to do so<br>4. Because I had an experience that I was sick after I missed dialysis/ I was hospitalized after I missed dialysis<br>5. I don't think following the dialysis schedule is very important to me<br>6. Other (Specify) ………………
23	Why do you think it is important to take your medicines as scheduled? (Please choose one best answer that applies to you.)	1. Because I fully understand that my kidney condition requires to take medicines as scheduled<br>2. Because taking medicines is important to keep my body healthy<br>3. Because a medical professional (my doctor, nurse, dietician, or other medical staff) told me to do so<br>4. Because I had an experience that I was sick after I missed medicines<br>5. Because I had an experience that I was hospitalized after I missed medicines<br>6. I don't think taking medicines is very important to me<br>7. Other specify : ……………	1. Because I fully understand that my kidney condition requires to take medicines as scheduled<br>2. Because taking medicines is important to keep my body healthy<br>3. Because a medical professional (my doctor, nurse, dietician, or other medical staff) told me to do so<br>4. Because I had an experience that I was sick after I missed medicines/ I was hospitalized after I missed medicines<br>5. I don't think taking medicines is very important to me<br>6. Other specify : ……………
33	Why do you think it is important for you to limit your fluid intake? (Please choose one best answer that applies to you.)	1. Because I fully understand that my kidney condition requires limiting fluid intake<br>2. Because limiting fluid intake is important to keep my body healthy<br>3. Because a medical professional (my doctor, nurse, dietician, or other medical staff) told me to do so<br>4. Because I got sick after I drank lots of fluid<br>5. Because I was hospitalized after I drank lots of fluid<br>6. I don't think limiting fluid is very important to me<br>7. Other (Specify):………………	1. Because I fully understand that my kidney condition requires limiting fluid intake<br>2. Because limiting fluid intake is important to keep my body healthy<br>3. Because a medical professional (my doctor, nurse, dietician, or other medical staff) told me to do so<br>4. Because I got sick after I drank lots of fluid/ I was hospitalized after I drank lots of fluid<br>5. I don't think limiting fluid is very important to me<br>6. Other (Specify):………………..

42	Why do you think it is important for you to watch your diet daily? (Please choose one best answer that applies to you.)	1. Because I fully understand that my kidney condition requires to watch my diet<br>2. Because watching my diet is important to keep my body healthy<br>3. Because a medical professional (my doctor, nurse, or dietician) told me to do so<br>4. Because I got sick after eating certain food that I was not supposed to eat<br>5. Because I was hospitalized after eating certain food that I was not supposed to eat<br>6. I don't think watching my diet is important to me<br>7. Other (Specify) : ……………	1. Because I fully understand that my kidney condition requires to watch my diet<br>2. Because watching my diet is important to keep my body healthy<br>3. Because a medical professional (my doctor, nurse, or dietician) told me to do so<br>4. Because I got sick after eating certain food that I was not supposed to eat/ I was hospitalized after eating certain food that I was not supposed to eat<br>5. I don't think watching my diet is important to me<br>6. Other (Specify) : ………………
27	What was the main reason for not taking your prescribed medicines past week?	1. Not applicable: I did not miss my medications<br>2. Forgot to take medications<br>3. Forgot to order medications<br>4. Cost of medications<br>5. Inconvenience<br>6. I was hospitalized<br>7. Side effects (Go to question28)<br>8. Other:	1. Not applicable: I did not miss my medications<br>2. Forgot to take medications/ order medications<br>3. Cost of medications<br>4. I was hospitalized<br>5. There was no one to assist me<br>6. Side effects (Go to question 28)<br>7. Other:
28	Answer this question if you have marked the above question as "Side effects."). What kind of side effect(s) to the medication(s) did you have? (Please choose the best answer that applies to you.)	1. Loss of appetite<br>2. Nausea/vomiting/diarrhea/constipation<br>3. Abdominal pain<br>4. Dizziness<br>5. Headache<br>6. Itching/skin problems<br>7. Other (Please specify symptoms):	1. Loss of appetite<br>2. Nausea/vomiting<br>3. Diarrhea/constipation<br>4. Abdominal pain<br>5. Dizziness<br>6. Headache<br>7. Itching/skin problems<br>8. Other (Please specify symptoms):
31	During the past week, how often have you followed the fluid restriction recommendations?	1. All of the time<br>2. Most of the time<br>3. About half of the time<br>4. Very seldom<br>5. None of the time	1. All of the time<br>2. Most of the time<br>3. About half of the time<br>4. Very seldom<br>5. None of the time/Can't remember
36	If you had difficulty following your fluid restriction recommendations, what type of	1. No difficulty<br>2. Not interested<br>3. I was unable to control fluid intake	1. Not interested<br>2. I was unable to control fluid intake<br>3. I don't understand how to limit fluid intake

	difficulty have you had?	4. I don't understand how to limit fluid intake<br>5. Other:………………	4. I feel faintishness when control my fluid intake<br>5. I can’t eat without having fluid<br>6. Other:…………
45	What type of difficulties have you had keeping your dietary recommendations?	1. Not applicable (No difficulty)<br>2. I was not willing to control what I want to eat<br>3. I was unable to avoid certain food products that were not recommended.<br>4. I don't understand the type of meal plan should follow<br>5. Other (Please specify):	1. Not applicable (No difficulty)<br>2. I do not have appetite to eat<br>3. I am not willing to control what I want to eat<br>4. I was unable to avoid certain foods that were not recommended.<br>5. I don't understand the type of meal plan should follow<br>6. Other (Please specify):
