# Supplementary material for: Psychometric properties of End Stage Renal Disease-Adherence Questionnaire-Sinhalese version among patients receiving haemodialysis
Source: PLoS One. 2023 Oct 20;18(10):e0292938. doi: 10.1371/journal.pone.0292938 (PMC10588851; doi:10.1371/journal.pone.0292938)
Supplement: Attachment — Submitted filename: Responses to Reviewers.docx [file pone.0292938.s003.docx]

**Responses to the reviewers’ comments**

**Title of the manuscript - Psychometric properties of End Stage Renal Disease-Adherence Questionnaire-Sinhalese version among patients receiving haemodialysis’**

We greatly appreciate the reviewers’ interest about the above titled manuscript and quality comments and suggestions offered by the reviewers for its further improvement. The observations of the reviewers gave a greater value to the manuscript and enhanced its validity and readability. The authors’ responses for the comments are given below. Thank you very much.

**Reviewer 1**

| **Reviewer comment** | **Author response** |
| --- | --- |
| **1**. **Adaptation of the questionnaire** Even though the process of translation and cultural adaptation have been well detailed in the Methodology section, nothing with regard to the qualitative components in these steps have been mentioned in the Results section. What this means is, the possible changes that had to be made to the questionnaire at each step before arriving at the final version, and why those were made. This is very important, especially, from a cultural adaptation standpoint. For example; it was noted that in SINESRD-AQ the answer options in questions 12. 23, 33 and 42 had been modified by combining the ‘got sick’ and ‘hospitalized’ options from the original ESRD-AQ. It would be important to specify, in the paper, such changes and reasons behind them. This modification has resulted in changes to the possible scores of the items and subscales. Similarly, changes in wording must have been required to make it suitable for the Sri Lankan setting. | The study did not involve any qualitative data collection method such as cognitive interviews in cultural adaptation process of the instrument in order to change or remove the items since original authors of English version of ESRD-AQ requested not doing any major changes to the questionnaire items or the format of the questionnaire. However, the minor changes that have been made to the questionnaire during the cultural adaptation and modified Delphi technique (while assessing face and content validity) are given in the table-(S2 File: Cultural adaptation of SINESRD-AQ).  During evaluation of face validity, based on the participant responses, a minor modification of the responses was done in the item 12, 23, 33 and 42 by combining the two responses ‘Because I had an experience that I was sick after I missed the particular treatment (dialysis, medications, fluid restrictions or prescribed diet) / I was hospitalized after I missed the particular treatment’. All the participants who responded as they missed the particular treatment, mentioned that they were hospitalized following the sickness. Therefore, both responses are applicable to such patients.  Moreover, according to the original study, question no. 12, 23, 33 and 42 are non-scored and recommended to analyse responses using descriptive statistics (Kim et al., 2010). The studies conducted later in other countries used numerical score to generate the final score of attitude/ perception subscale (question no. 12, 23, 33 and 42 are assessed knowledge/attitude of the patient on particular treatment. These questions are scored separately from the other perception questions of the attitude/perception subscale -11, 22, 32, 41). A similar method was used in the present study to obtain the total score in the attitude/ perception subscale separately for knowledge/attitude questions and perception questions. Since there is no originally created and uniform scoring for the given questions, it did not affect the total score of the scale in the present study. |
| **2. Utilization of the findings of the CFA** Based on the CFA findings, the authors have proposed that a 5-item 2-factor model is the best fit for Adherence Behaviour subscale, and 8-item 2-factor is most appropriate for Attitude/Perception subscale. However, this has not resulted in the authors making (or at least suggesting) any changes to the final questionnaire or the way it should be scored. Since each item has its individual importance, removing them based on the CFA interpretation may not be appropriate in this type of questionnaire. But, when a scale score is calculated, the items that should be used and the scoring method may have to be modified in the face of such findings. | The study only used CFA to test the pre-defined relationships within the data, while comparing different models to determine the right fit for particular data set. It was not attempted to remove or modify any of the item from the questionnaire by the authors as each item of the given subscales holds a specific importance in managing CKD among patients. Therefore, it was also not involved in changing the scoring method of the original scale, especially direct adherence behaviour subscale. However, as question no. 17 and 18 show negative factor loadings, it indicates that wrong interpretation of the question by the participants or less importance of the item in assessing positive adherence among patients in Sri Lankan hospitals. However, we didn’t remove these items from the questionnaire considering the originality of the questionnaire and its’ clinical importance. |
| **3. Presence of negative factor loadings in** **Adherence Behaviour subscale** All the items assessing adherence in SINESRD-AQ were coded to have higher values for higher adherence. Yet, it is interesting to note that factor loadings of all the CFA models are negative for items related to shortening HD sessions (items 17 and 18). If we assume that the latent factor is for positive treatment adherence, HD session shortening is acting in the opposite direction. This seems counter intuitive. Even though I am not experienced in practicalities of HD session in Sri Lanka, my suspicion is that patients have little or no say in determining the length of sessions. Considering the authority of health staff in the Sri Lankan healthcare setup, these questions could be irrelevant or incorrectly interpreted by the patients. | In the present study, most of the patients’ HD sessions were shortened due to experiencing the side effects during the HD treatment, e.g. muscle cramping, low or high blood pressure or due to a problem in AVF/vascular access. In the original study, shortening of the dialysis session was considered without having any medical reasons. Accordingly, in the present study, only a very few patients mentioned that they shortened their HD session due to a personal reason (5.3%) and most of them mentioned that they shortened the HD session only twice or less in the past month (4%) for less than 30 minutes. Therefore, there can be little or no impact of shortening the dialysis session on overall positive treatment adherence of the patients in the present study.  Based on the findings, negative factor loadings of item no.17 and 18 indicate when the frequency and duration of shortening of the dialysis treatment increased, the adherence to haemodialysis among participants was good.  Patients might be happy with shortened dialysis sessions when experiencing side effects or when having another personal reason to discontinue from the dialysis machine. These patients might have shown good adherence to the other modalities of haemodialysis treatment such as attendance to haemodialysis, adherence to medication, fluid and diet restrictions. However, we didn’t remove the questions from the questionnaire as per the request of original authors not to remove or change the items in the original ESRD-AQ.  Also, as the question is based on last month, patients might not have given the correct answer for this question due to recall bias. |
| **4. Rationale of using factor analysis in a questionnaire such as this** It seems like a bold decision by the authors to utilize factor analysis for this type of questionnaire, while it had not been used in any of the previous publications on ESRD-AQ given as references. As noted in the manuscript itself, the original paper by Kim et al. (2010) notes that the instrument would not be suitable to assessed using a measure of internal consistency such as Cronbach’s Alpha. Then again, it uses the summing of item scores to produce subscale scores, which suggests the presence of an underlying common construct that is being measured by the selected items. Factor analysis assumes the presence of latent variables (factors) represented by one or more of the scale items. Considering that each item of SINESRD-AQ measures a specific aspects of CKD management, the need of factor analysis in this study could be questioned. | CFA was used only to identify how each item theoretically related to their respective subscale (latent construct/factor). Since many previous publications have clinically validated this instrument (using known-groups analysis), authors completely agree that there is no need to conduct factor analysis to validate this type of questionnaire. However, in the present study, authors attempted to show that the given two subscales are valid not only clinically but also theoretically after performing CFA.  Since the original study has not assessed internal consistency reliability (Cronbach’s Alpha), the present study also omitted it from the analysis. Instead present study evaluated test-retest reliability of both subscales separately. We have removed convergent and discriminant validity findings from the results since these findings do not add any value in modifying or changing the items or scoring of the questionnaire. |
| **5**. **Uniformity on usage of the second subscale name** The second subscale mentioned in the paper seem to take two forms: Attitude/Perception and Attitude/Knowledge. But it must be stated that this error was seen in the original paper by Kim et al. (2010) as well. I would suggest sticking to one of the two, preferably Attitude/Perception, as knowledge does not seem to be what the questions measure. | Second subscale name was stated in all the relevant places as ‘attitude/perception subscale”. |
| **6. Reclassification of item responses** In this, the focus is on lines from 255 to 260 (on pages 12 and 13) as well as Table 2 on page 14. It has not been explained how these percentages were arrived at. What does it mean to have 96.7% HD adherence when the relevant question (14) only has answers on the number of HD treatments that were missed? Similarly, for the other values noted in page 12 and Table 2. There seem to have been a reclassification of responses performed. But this has not been mentioned in the methodology or results section. | Description related to descriptive statistics analysis of both adherence behaviour subscale and attitude/perception subscale is included in the Methodology section under subheading “Measurements”. Page No. 9-10 (Line 188-208) |
| **7. Errors in writing** Even though the overall writing of the manuscript is of good quality, there are a fair amount of errors that have to be corrected. It would be difficult to point out each. A proper and detailed proofreading would be required. Some of them are noted below: • **Lines 51 to 53 on page 3** – This sentence is not clear. Perhaps stating it as “… 2003 to 2013 had increased by over eight thousand per year with around thousand haemodialysis sessions per month in 2013”. • **Lines 84 to 86 in page 4 and 5** – This is an improperly structured sentence. • **Line 105 on page 5** – Shouldn’t it be “CKD of unknown origin”? • **Line 125 on page 6** – Shouldn’t it be “A committee consisting of…”? • **Line 249 on page 12** - up to ordinary level could mean any level of education up to that level. Shouldn’t this be Grade 5 up to Ordinary Level? | - **Lines 51 to 53 on page 3** –   Rewrote the sentence as ‘The number of haemodialysis sessions carried out in National Hospital of Sri Lanka was dramatically increased from approximately 4600 in 2003 to more than 11,600 in 2013 with an average of thousand haemodialysis sessions per month’ (Page 3)   - **Lines 84 to 86 in page 4 and 5**   Rewrote the sentence as ‘However, there is limited evidence available on self-reported adherence among patients receiving haemodialysis in Sri Lankan hospitals’. (Page 4)   - **Line 105 on page 5** – Shouldn’t it be “CKD of unknown origin”?   Corrected as ‘CKD of unknown origin’ (Page No.5)   - **Line 125 on page 6** – Shouldn’t it be “A committee consisting of…”?   Corrected as ‘A committee consisting of experts in questionnaire designing…’ (Page No.6)   - **Line 249 on page 12**   Included explanation for ‘up to ordinary level’  as ‘grade 6 to grade 11 in school’ (Page No. 13) |
| **8. Errors in references** There were few mistakes in referencing of the manuscript. It would be good to recheck them even if a referencing software was used. Some of the noted errors are: • **Line 75 on page 4** – Even though Palestine is mentioned, the references does not seem to give results from a Palestinian study. • **Line 149 on page 7** – the reference 17 appears twice. It seems that the second one should be 18, as the next reference noted in the text is 19. • **Line 202 on page 10** – Reference 3 does not seem to be relevant here. Order of references should be 8, 9, 10, … **• Lines 534 and 535 on page 28** – The reference is incomplete  • **Line 541 on page 28** – Mixing up of page number and DOI  • **Lines 570 to 573 on pages 29 and 30** – Some components in full capital  • **Line 575 on page 30** - incomplete reference | - **Line 75 on page 4**   References were checked and corrected, included reference number 6 which is a Palestinian study. (Line 75, page No. 4)   - **Line 149 on page 7**   In-text reference 18 is included for both I-CVI and Modified Kappa statistic coefficient (k) cut-off values. (Page No.7 Line 149)   - **Line 202 on page 10**   Reference checked and removed reference 3. Order corrected as 8,9,10,11,12 (Page No.11, Line 228)   - **Lines 534 and 535 on page 28**   Completed reference included. (Page No.29, Line 561-562)  Lynn MR. Determination and quantification of content validity. Nursing research. 1986 Nov. 35 (6): 382-385. <https://doi.org/10.1097/00006199-198611000-00017>   - **Line 541 on page 28**   Corrected the reference as follows (Page No.29, Line567-569)  Shrotryia VK, Dhanda U. Content validity of assessment instrument for employee engagement. Sage Open. 2019 Feb;9(1):1-7. <http://dx.doi.org/10.1177/2158244018821751>   - **Lines 570 to 573 on pages 29 and 30**   Rewrote the reference (Line 596-599, Page No.31)  Malete L, Ricketts C, Kim S, Tshube T, Mphela T, Adamba C, & Ocansey R. An Examination of the Latent Structure and Reproducibility of the Life Skills Scale for Sport in Botswana and Ghana. Frontiers in psychology. 2022 May 1; 13. <https://doi.org/10.3389/fpsyg.2022.858406>   - **Line 575 on page 30**   Included completed reference (Page No.31, Line 602-603)  Kaiser HF. A second-generation little jiffy. Psychometrika. 1970;35(4). 401-415. <http://dx.doi.org/10.1007/BF02291817> |

**Reviewer 2**

| **Reviewer comment** | **Author response** |
| --- | --- |
| 1. Line 49 //Nonetheless, haemodialysis has become as the most efficient and practical treatment modality among patients with ESRD in Sri Lanka due to resource constraints and shortage of kidney donors for transplantation [4]// Referred article does not discuss haemodialysis being the most efficient treatment for ESRD. Please correct. | Corrected as follows.  Nonetheless, haemodialysis has become as the most demanding and practical treatment modality among patients with ESRD in Sri Lanka due to resource constraints and shortage of kidney donors for transplantation [4]. |
| 2. Line 51 //It was observed that the number of dialysis sessions carried out in National Hospital of Sri Lanka from 2003 to 2013 were increased approximately over eight thousand with thousand haemodialysis sessions per month// Does this refer to haemodialysis or all types of dialyses together? | It only refers to haemodialysis sessions carried out in National Hospital of Sri Lanka  The sentence was corrected as follows.  The number of haemodialysis sessions carried out in National Hospital of Sri Lanka was dramatically increased from approximately 4600 in 2003 to more than 11,600 in 2013 with an average of thousand haemodialysis sessions per month [5]. |
| 3. Line 152 //The resulting version was administered to a purposively selected group of patients (n=10) receiving haemodialysis to evaluate face validity// Please explain which characteristics of the participants were considered in ‘purposively selection’ and why those characteristics were considered. | The characteristics of the participants involved in evaluating face validity of SINESRD-AQ were as follows.  Diagnosed patients with CKD undergoing haemodialysis (ESRD) admitted to selected  government hospital haemodialysis units in Colombo district, Sri Lanka during the study  period.  Those who received haemodialysis treatment for more than six consecutive months according to a routine haemodialysis schedule for at least one session per week.  Patients who were older than 18 years of age and able to understand or speak  Sinhala, English or Tamil language  Maximum variation sampling was considered considering age, gender, duration of CKD and undergoing haemodialysis and income level. |
| 4. Line 187 //data were collected using interviewer administered SINESRD-AQ// Please explain why the study instrument was administered through an interviewer, when it’s designed to be used as self-reported tool. | Eventhough the original ESRD-AQ designed to use as self-administered questionnaire, considering the clinical condition and patients’ difficulties in reading and answering the questionnaire during their HD treatment, authors decided to administer ESRD-AQ as an interviewer administered questionnaire. Therefore, the questions that are difficult to understand by the participants were clearly explained by the principal investigator during the data collection. It helped to eliminate most of the recall bias, wrong interpretations and poor concern and attention of the patients during data collection. |
| 5. Line 379 How do you justify using Cronbach’s Alpha to assess internal consistency of the translated study instrument when the original instrument has omitted it due to instrument design does not consist homogenous items to measure internal consistency reliability? | Since the original study has not assessed internal consistency reliability (Cronbach’s Alpha), the present study also omitted that reliability analysis. Instead, present study evaluated test-retest reliability of both subscales separately. |
| 6. Number of tables - Please check with journal guidelines for the number of tables/ figures allowed. | The author guidelines of the journal have not provided specific number of tables to be included in the manuscript. |
